# External Beam Radiotherapy of Recurrent Glioma: Radiation Tolerance of the Human Brain

**DOI:** 10.3390/cancers4020379

**Published:** 2012-04-05

**Authors:** Peter Sminia, Ramona Mayer

**Affiliations:** 1 Department of Radiation Oncology, Radiobiology Section, VU University Medical Center, De Boelelaan 1117, P.O. Box 7057, Amsterdam, The Netherlands; 2 EBG MedAustron GmbH., Viktor Kaplan-Strasse 2, A-2700, Wiener Neustadt, Austria; E-Mails: ramona.mayer@medaustron.at (R.M.)

**Keywords:** equivalent total dose (EQD2), re-irradiation, brain, glioma, late side effects

## Abstract

Malignant gliomas relapse in close proximity to the resection site, which is the postoperatively irradiated volume. Studies on re-irradiation of glioma were examined regarding radiation-induced late adverse effects (*i.e*., brain tissue necrosis), to obtain information on the tolerance dose and treatment volume of normal human brain tissue. The studies were analyzed using the linear-quadratic model to express the re-irradiation tolerance in cumulative equivalent total doses when applied in 2 Gy fractions (EQD2_cumulative_). Analysis shows that the EQD2_cumulative_ increases from conventional re-irradiation series to fractionated stereotactic radiotherapy (FSRT) to LINAC-based stereotactic radiosurgery (SRS). The mean time interval between primary radiotherapy and the re-irradiation course was shortened from 30 months for conventional re-irradiation to 17 and 10 months for FSRT and SRS, respectively. Following conventional re-irradiation, radiation-induced normal brain tissue necrosis occurred beyond an EQD2_cumulative_ around 100 Gy. With increasing conformality of therapy, the smaller the treatment volume is, the higher the radiation dose that can be tolerated. Despite the dose escalation, no increase in late normal tissue toxicity was reported. On basis of our analysis, the use of particle therapy in the treatment of recurrent gliomas, because of the optimized physical dose distribution in the tumour and surrounding healthy brain tissue, should be considered for future clinical trials.

## 1. Introduction

Gliomas are the most common primary brain tumours, with glioblastoma multiforme (GBM) being the most frequent, aggressive and invasive tumour type. Postoperative radiotherapy with concomitant temozolomide (TMZ) has become the standard of care for patients with newly diagnosed GBM, based on the results of a large European-Canadian phase III trial [1]. This latter randomised trial demonstrated a significant increase in median survival from 12.1 months after radiotherapy alone to 14.6 months after radiotherapy combined with TMZ. Benefits of TMZ with radiotherapy lasted throughout 5 years of follow-up, with a survival rate of 9.8% *versus* 1.9% after radiotherapy alone [2]. Despite this important success, most patients die from recurrent disease. The high recurrence rate of about 100% is due to the infiltrative growth characteristics of this tumour type, with its spread throughout normal brain tissue, and high resistance to both radiotherapy and chemotherapy.

 Malignant gliomas relapse in up to 90%, in close proximity to the resection site or the initially irradiated volume [3]. Treatment options for recurrent glioma remain limited and include re-resection, chemotherapy, and a second course of radiotherapy. However, there is no standard protocol for re-irradiation of brain tumours. The limited radiation tolerance of normal brain tissue determines the re-irradiation dose that can be applied in addition to the dose of the initial irradiation course, with an acceptable late morbidity profile. A large variety of palliative re-irradiation treatment schemes are reported, with different total dose, number and size of fractions. Retreatment schemes for recurrent gliomas often comprise hypofractionation as well as additional therapy, mostly anti-angiogenic drugs. Generally, the applied re-irradiation technique is chosen based on tumour volume. Only tumour recurrences that are sufficiently small can be treated with high conformality and allow the use of hypofractionated or single dose treatment; this spares normal tissue, which decreases the risk of volume-dependent late toxicity [4,5].

 This paper presents an overview of current clinical data on re-irradiation of recurrent glioma with respect to the tolerance dose of normal, healthy brain tissue. To obtain the cumulative radiation dose from the initial and the re-irradiation protocols and to enable comparison of data between studies, rather than taking the “physical” dose, the tolerance dose of normal brain tissue is presented as a ‘biological’ equivalent total dose when applied in 2 Gy fractions (EQD2), estimated by analysis using the linear quadratic model [6,7,8]. Such analysis provides insight into the re-irradiation tolerance of the normal, healthy brain that might be used as a guideline in clinical practice. Particle irradiation has a beneficial dose distribution and should be investigated in the small proportion of patients with small and well described recurrent glioma.

## 2. Results

Re-irradiation studies are summarized according to conventional radiotherapy (Table 1), fractionated stereotactic radiotherapy (FSRT) (Table 2) and LINAC-based stereotactic radiosurgery (SRS) (Table 3). Details are provided on histology, time interval between the primary and re-irradiation courses, physical median dose and number of fractions of both irradiation courses, as well as their EQD2 values. Data on patient survival and the probability of side-effects are also shown. Only a few studies are restrictive and investigate a distinct histological subtype, while the other series include a mixture of different histological subtypes with relatively small numbers of patients (range 10–172). With regard to the “treatment volume”, different definitions are used. In most FSRT and SRS series (Table 2 and Table 3), the planning target volume (PTV) is reported, generally including the CT/MR contrast-enhancing tumour with a safety margin ranging from 2 mm to 1 cm.

Data on re-irradiation with conventional external beam radiotherapy are given in Table 1 [9,10,11,12,13,14,15]. The cumulative EQD2 ranged from 81.6 to 102.8 Gy (mean ± S.D: 92.6 ± 6.8 Gy; n = 11) (Table 1). In these series, no information on the PTV was provided. The mean time elapsed between primary radiotherapy and re-irradiation was 29.9 ± 14.1 months (range 14–55 months; n = 7). Acute neurological toxicity (9%) and radionecrosis (6%) were reported in one series of patients treated with a relatively low EQD2_cumulative_ (87.7 Gy); however, in a twice daily regimen [12]. In contrast, Veninga *et al*. [13] observed late effects only in those patients receiving a EQD2_cumulative_ of >102 Gy.

Data on re-irradiation series using LINAC-based FSRT are given in Table 2 [16,17,18,19,20,21,22,23,24,25,26,27,28,29,30,31]. One exception is the study of Kohshi *et al*. [24], in which FSRT was delivered with a gamma unit using a noninvasive fixation system.

The cumulative EQD2 ranged from 86.1–133.9 Gy (mean ± S.D: 109.9 ± 13.8 Gy; n = 16) (Table 2). The mean time interval between initial radiotherapy and reirradiation was 16.7 ± 11.1 months (range 3–48 months; n = 17). The mean PTV was 27.6 ± 11.9 cc (range 8.7–51.1 cc; n = 16). Radiochemotherapy with Paclitacel was performed in all 88 patients reported by Lederman *et al*. [19]. Grosu and coworkers treated 29 of 44 patients with temozolomide [21]. Severe acute radiation-induced toxicity of 8% of the patients was reported in one study (EQD2_cumulative_ of 99.2 Gy) [17]. In some but not all series, with FSRT, pathologically confirmed radionecrosis was reported in ~2–12% of patients irradiated at EQD2_cumulative_ > 96 Gy (Table 2). Analysis of 16 studies shows that the incidence of radiation necrosis was not correlated with the EQD2_cumulative_ or with the time interval between the initial radiation and re-irradiation course.

The LINAC-based SRS re-irradiation data are summarized in Table 3 [17,31,32,33,34,35,36]. The mean time interval between the two courses of radiotherapy was 10.4 ± 1.2 months (range 9.1–12.5 months; n = 6). The mean irradiation volume was 16.9 ± 7.9 cc (range 10–30 cc; n = 7), which is smaller than with conventional re-irradiation or FSRT. The cumulative EQD2 ranged from 111.6 Gy to ~150 Gy (mean ± S.D, 130.5 ± 13.5 Gy; n = 7) (Table 3). Radionecrosis, up to an incidence of 17%, was reported in two studies after an EQD2_cumulative_ of >137 Gy [17,31]; no additional chemotherapy was administered in these two studies.

**Figure 1:**
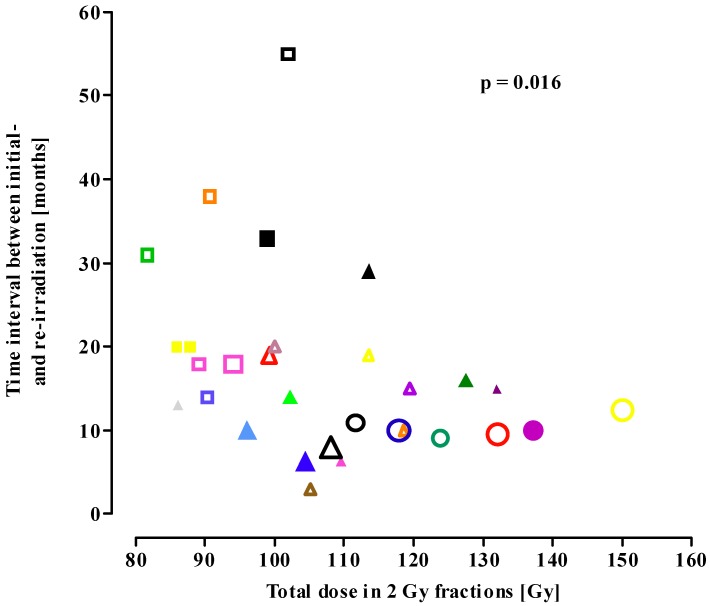
Total dose in 2 Gy fractions (EQD2_cumulative_) as a function of the time interval between initial treatment and conventional re-irradiation (squares), fractionated stereotactic radiotherapy (triangles) and stereotactic radiosurgery (circles). Open symbols: none of the patients in the study showed brain necrosis; solid symbols: patient(s) with radionecrosis in the study. Symbol size represents the number of patients in the study. Small-sized symbol <25 patients; median-sized symbol 26–50 patients; large-sized symbol >50 patients. Symbols in the figure match the symbols used in Table 1, Table 2, Table 3. (Spearman nonparametric correlation: *p* = 0.016).

Figure 1 shows the influence of the time interval from initial radiotherapy to reirradiation and the EQD2_cumulative_ on the incidence of radionecrosis. The EQD2_cumulative_ increased from conventional re-irradiation series (squares) to FSRT (triangles) to SRS series (circles), with a concomitant decrease in the mean time interval between the initial irradiation course and re-irradiation. Despite the higher biological dose and shorter time interval, the incidence of radionecrosis did not differ between the three re-irradiation procedures (Figure 1). A significant correlation (*p* = 0.016) was found: the higher the EQD2_cumulative_, the shorter the time interval between the initial exposure and re-irradiation.

**Table 1:** Clinical data on brain re-irradiation by conventional radiotherapy: Physical dose and equivalent total dose in 2 Gy fractions (EQD2), survival and toxicity.

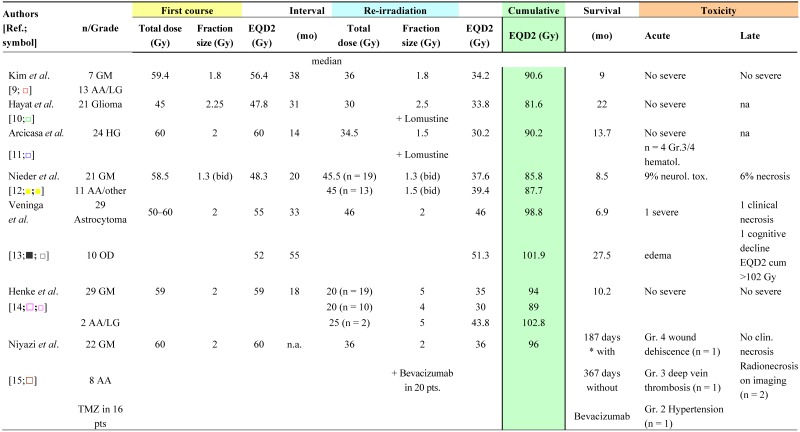

GM: Glioblastoma multiforme; AA: Anaplastic astrocytoma; bid: twice a day; n.s.: not stated.

**Table 2:** Clinical data on brain re-irradiation by fractionated stereotactic radiotherapy: physical dose and equivalent total dose in 2 Gy fractions (EQD2), survival and toxicity.

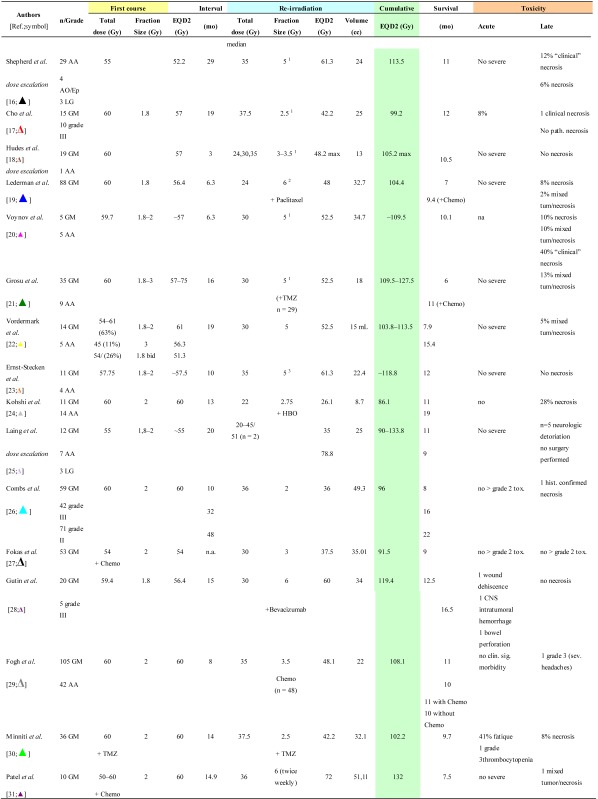

^1^: 5 days a week; ^2^: once a week; ^3^: three times a week; nr = not reached; AA: Anaplastic astrocytoma; AO: Anaplastic oligodendroglioma; GM: Glioblastoma multiforme; OD: Oligodendroglioma; LG:Low grade glioma.

**Table 3:** Clinical data on brain re-irradiation by stereotactic radiosurgery: physical dose and equivalent total dose in 2 Gy fractions (EQD2), survival and toxicity.

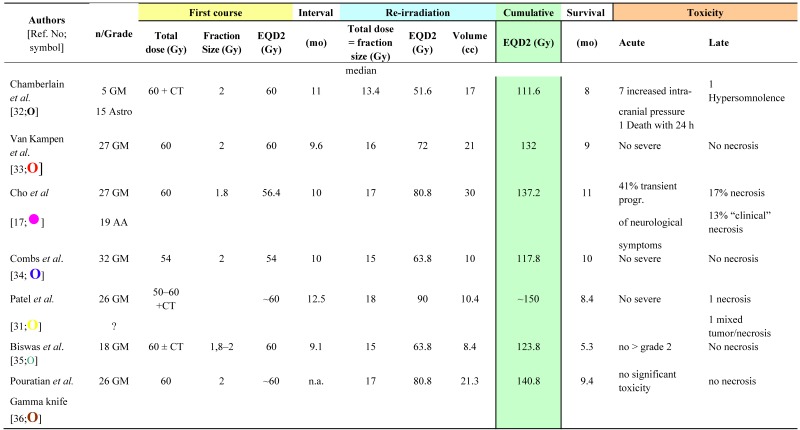

**Figure 2:**
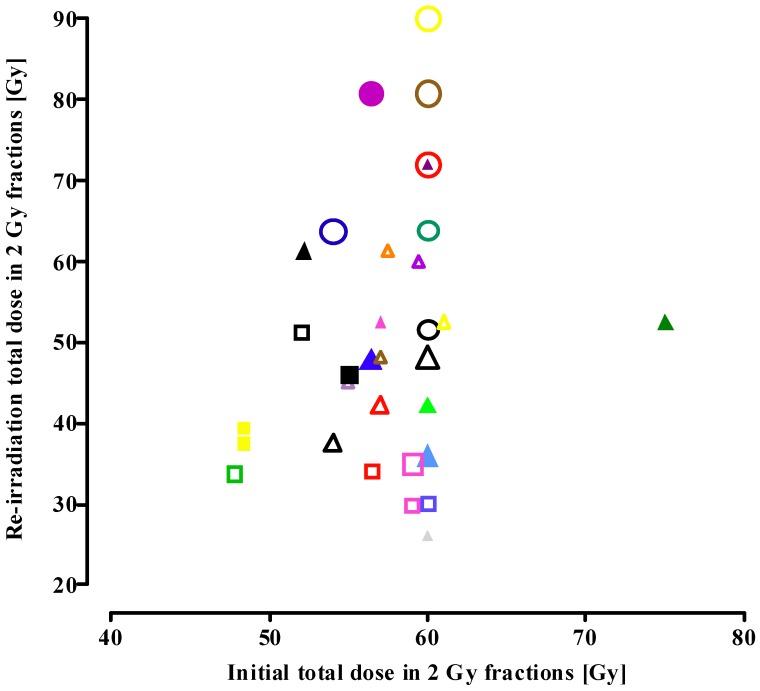
Correlation of the initial dose (EQD2_initial_) and re-irradiation dose (EQD2_reirradiation_) for patients re-irradiated with conventional radiotherapy, fractionated stereotactic radiotherapy and stereotactic radiosurgery (see legend to Figure 1 for an explanation of the symbols).

Data on the correlation between the EQD2 of the initial scheme and of the re-irradiation scheme are presented in Figure 2. In patients re-irradiated with conventional radiotherapy, the EQD2_re-irradiation_ was always lower than the EQD2_initial_ (squares).

In contrast, in FSRT series the EQD2_re-irradiation_ was higher than the EQD2_initial_ in four out of 16 studies (triangles). One exception (lowest triangle, Figure 2) is the study [24] using re-irradiation in combination with hyperbaric oxygen therapy, resulting in radionecrosis at a relatively low EQD2_re-irradiation_. In studies using SRS for retreatment of gliomas, the EQD2_re-irradiation_ exceeded the EQD2_initial_ in all but one of the seven series (circles, Figure 2).

**Figure 3:**
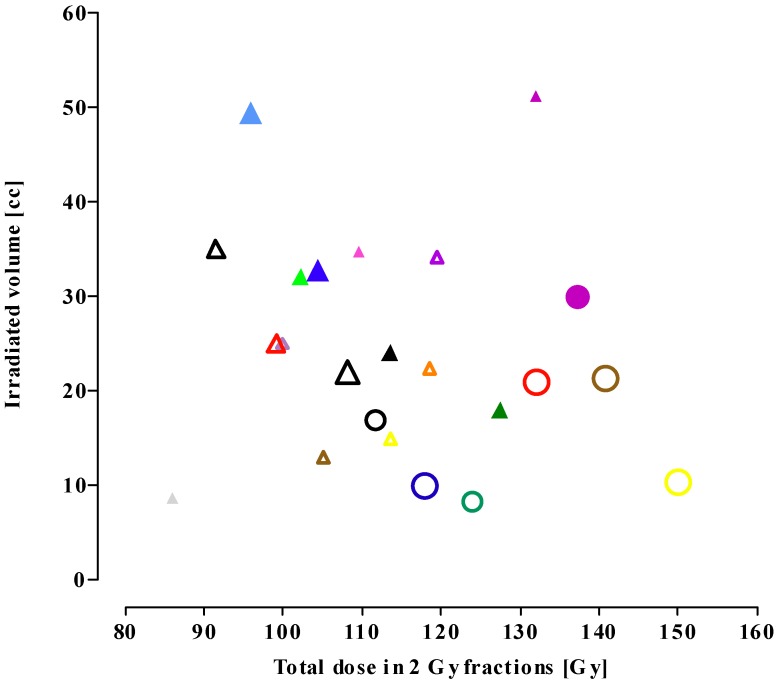
Treatment volume *versus* cumulative dose in 2 Gy fractions (EQD2_cumulative_). The symbols match the studies presented in Table 2 and Table 3 (see legend to Figure 1 for an explanation of the symbols).

Figure 3 shows correlations between the EQD2_cumulative_ and the irradiated volume. The figure shows a decrease in treatment volume from FSRT (triangles) re-irradiation series to SRS series (circles). The smaller the re-irradiation volume, the higher the re-irradiation dose applied.

Table 4 presents a summary of the current treatment procedures and treatment data; it shows that increasing conformality of the re-irradiation technique allows exposure to a smaller treatment volume and a higher cumulative biological dose, with a shorter time interval after initial irradiation.

**Table 4:** Data on current re-irradiation protocols for the human brain, showing the EQD2_cumulative_, time interval between the initial radiotherapy course and re-irradiation course, and treatment volume (values are mean ± SD (range); n = 6–17).

Re-irradiation procedure	EQD2_cumulative_ [Gy]	Time interval between initial radiotherapy and re-irradiation [months]	Mean treatment volume [cc]
Conventional radiotherapy	92.6 ± 6.8 (81.6–102.8)	29.9 ± 14.1 (14–55)	No data
Fractionated stereotactic radiotherapy	109.9 ± 13.8 (86.1–133.9)	16.7 ± 11.1 (3–48)	27.6 ± 11.9 (8.7–51.1)
Stereotactic radiosurgery	130.5 ± 13.5 (111.6 to ~150)	10.4 ± 1.2 (9.1–12.5)	16.9 ± 7.9 (8.4–30)

## 3. Discussion

### 3.1. Re-Irradiation Tolerance of the CNS

The different re-irradiation protocols show distinct variability in the tolerance of the human brain with regard to the total irradiation dose when applied in 2 Gy fractions. This EQD2_cumulative_ value increases from conventional re-irradiation (81.6–102.8 Gy) to FSRT (86.1–133.9 Gy) and SRS (111.6 to ~150 Gy). The incidence of radionecrosis increased to approximately 17%, but was independent of the chosen re-irradiation technique and EQD2_cumulative_. The EQD2 values were calculated according to the linear-quadratic formula, which is the generally accepted standard model for dose-fractionation analyses [7] in clinical radiotherapy. However, in fractionated stereotactic re-irradiation, fraction sizes mostly exceed 5 Gy, and in radiosurgery single-dose fractions as high as 18 Gy are applied. The validity of the linear-quadratic model for such high-dose fractions is questionable and estimates should be considered carefully [37,38]. The model has been described to either *overpredict* or *underestimate* the biological effect of high single-dose fractions. In particular, in the high single-dose range the model might require refinement [8]. Additional to the fraction size, kinetics of sublethal DNA damage repair, which is considered to be bi-exponential with a fast and a slow repair component, could determine the biological dose to the healthy brain. Both the number of beams and the time interval between their application and the protracted treatment time might result in a *lower* biological dose to the normal brain due to *fast* repair kinetics. A *higher* biological dose on the normal brain might explain toxicity in the one study using a hyperfractionation regimen, although this was not to be expected because of the time interval of 6 hours between the two daily fractions [12]. In this latter study, despite an EQD2_cumulative_ of <90 Gy, radionecrosis was reported, while no necrosis was reported in other conventional re-irradiation studies with higher EQD2_cumulative_. This observation indicates *slow* DNA damage repair, which is incomplete in the time interval of 6 hours between the two subsequent daily fractions, resulting in a *higher* EQD2 than predicted by complete repair model calculations. The repair half-time of normal brain tissue is not known, but long mono-exponential repair half-times in the order of 2.5 to 4 h for late morbidity were estimated from the CHART trial, in which head and neck cancer patients were treated with three fractions per day spaced 6 h apart [39]. In FSRT and SRT series radionecrosis were reported after a EQD2cumulative of ≥96 Gy and >137 Gy, respectively. An exception should be made for the study using hyperbaric oxygen as radiosensitizer, where radionecrosis was observed at a EQD2cumulative as low as 86.2 Gy [24]. In that study, recurrent glioma patients were treated with fractionated gamma knife irradiation within 7 min following hyperbaric oxygen therapy [24]. Despite a relatively low EQD2cumulative of 86 Gy in that study, the percentage of necrosis was relatively high, i.e., 28% (cf Table 2). This effect seems to be due to a radiosensitizing effect of oxygen on the normal nervous tissue, additional to tumour radiosensitization. For a discussion on this issue, we refer to Mayer and Sminia [40]. The present review on the re-irradiation tolerance of normal, healthy brain indicates that the following issues warrant more attention.

#### 3.1.1. Total Cumulative Dose

The mean cumulative dose (EQD2_cumulative_) was found to increase from 92.6 ± 6.8 Gy (mean ± S.D.; n = 11) in conventional re-irradiation series to 109.9 ± 13.8 Gy (mean ± S.D., n = 16) following FSRT to 130.5 ± 13.5 Gy (mean ± S.D., n = 7) in LINAC-based SRS. This increase in radiation dose was not reflected in an increase in the incidence of radionecrosis, which is likely due to a decrease in treatment volume (see point D).

#### 3.1.2. Time Interval between Initial Exposure and Retreatment

The time interval between the initial irradiation and retreatment ranged from 3–55 months (Figure 1; Table 1, Table 2, Table 3). Following the initial exposure tissue recovery will start, which is a time-dependent process. For the spinal cord, a morphologically similar nervous tissue, there is considerable experimental and clinical evidence for long-term recovery from occult radiation injury (e.g., [41,42]). The re-irradiation tolerance of the primate spinal cord increases progressively with increasing time interval between initial exposure and re-irradiation [41]. For standard fractionation schemes, a dose response relationship for brain necrosis following irradiation has been reported, with an incidence of necrosis of 5% and 10% after 72 Gy and 90 Gy, respectively, in 2 Gy fractions [5]. Comparison with data from the present analysis, showing ± a 15–40% higher cumulative tolerance dose for brain necrosis, supports long-term recovery from radiation injury for the human brain. However, our analysis does not show a correlation between the time interval (range 3–55 months) and tolerance to re-irradiation. For example, in the study with the shortest time interval of 3 months, an EQD2_cumulative_ of 105 Gy did not result in tissue necrosis [18], while in another report [21] necrosis was found at an even lower EQD2_cumulative_ and longer time elapsed since initial irradiation. Also, shortening of the mean time interval from 30 months for conventional re-irradiation to 17 and 10 months for FSRT and SRS, respectively, (Table 4) did not increase the probability of radiation-induced brain necrosis. These observations suggest a relatively fast process of (partial) long-term recovery, in the order of months rather than of years.

#### 3.1.3. Size of the Initial Dose

For the spinal cord, the re-irradiation tolerance was shown to be higher with a lower initially applied dose, indicating better recovery capacity [41,42]. Since the EQD2 of the primary radiation dose applied to glioma patients is generally ~60 Gy, *i.e*., the 30 fractions of a 2 Gy standard scheme or a biologically equivalent scheme, the present data (Table 1, Table 2, Table 3) do not allow to draw conclusions about this phenomenon.

#### 3.1.4. Treatment Volume

The present analysis shows that the actually prescribed re-irradiation dose increases with a change in irradiation technique from conventional to FSRT to LINAC-based SRS re-treatment (Figure 2). In patients re-irradiated with conventional radiotherapy, the EQD2_re-irradiation_ was always lower than the EQD2_initial_, whereas it was higher in some of the FSRT series and in 6 out of 7 SRS series. In the SRS studies, re-irradiation dose regimens were used that would likely exceed the tolerance dose of the brain. An inverse correlation was found between the cumulative radiation dose and treatment volume (Figure 3, Table 4). This is likely due to the choice for high conformal therapy in case of small tumour recurrence and a consequent decrease in late normal tissue toxicity. Thus, normal brain tissue shows a large volume effect in the clinically relevant dose range, *i.e*., the smaller the irradiated volume, the higher the tolerance dose. In a recent review [5], a clear correlation was reported between the maximal tolerated dose for radiation necrosis and the irradiation volume. For SRS, the volume of brain receiving ≥ 12 Gy was found to correlate with both the incidence of radiation necrosis and asymptomatic radiologic changes [5].

### 3.2. Possible Role of Particle Therapy

As a consequence of the inverse correlation between the irradiation tolerance of the human brain and the treatment volume, further reduction of the exposed volume of normal brain tissue inside the high-dose treatment area would permit dose escalation in the tumour target. Such an improved physical selectivity can be achieved using particle therapy. These particles exhibit an inverse dose profile during penetration, *i.e*., a rather small energy deposition in the entrance channel followed by an increase of the energy deposition and a steeply sloping decrease after reaching the maximum, the so-called Bragg peak. In addition lateral scattering effects are relatively small, especially for ions with higher masses. Protons, as already state-of-the-art particles in ion beam therapy, have a relative biological effectiveness (RBE) comparable to photons, *i.e*., 1.1. Carbon ions share the favourable physical properties of protons but have a biological advantage [43]. Their biological efficiency increases at the end of the beam’s range, while being low in the entrance channel. When different clinical situations are considered, the biological advantages of carbon ions in comparison to protons are expected to be most pronounced for tumours that demonstrate low radiosensitivity when treated with photons [44,45]. Local values for RBE can be as high as approximately 3 for carbon ions and depend on many factors, which have to be addressed during treatment planning.

One has to keep in mind that only small and well described recurrences can be considered as potential candidates of particle therapy. An interesting study has started at the Ion Therapy Center and the University Hospital in Heidelberg (Germany). In the Phase I/II CINDERELLA trial, re-irradiation using carbon ions is compared to FSRT applied to the area of contrast enhancement representing high-grade tumour areas in patients with recurrent gliomas [46]. In Phase I, the recommended dose of carbon ion radiotherapy will be determined in a dose escalation scheme. In Phase II (randomized), the recommended dose will be evaluated in the experimental arm, compared to the standard arm, using FSRT with a total dose of 36 Gy in single doses of 2 Gy. Primary endpoint of Phase I is toxicity, and of Part II is survival after re-irradiation at 12 months; the secondary endpoint is progression-free survival.

### 3.3. Neurocognitive Function after Re-Irradiation

With improving survival of glioma patients, focus on long-term treatment-related morbidity has increased, with the effect of brain (re-)irradiation on cognitive performance as major concern. Establishing the effect of radiation on patients’ neurocognitive impairment is difficult because of confounding factors like the tumour itself, surgery, chemotherapy, concurrent illnesses, neurologic co-morbidity and medications [47]. In their review, Laack and Brown conclude that high total dose, large fraction size and large brain volumes are associated with increased risk of neurocognitive decline after radiotherapy.

### 3.4. Survival Data Including Data with Additional Chemotherapy

When looking at survival data after re-irradiation (Table 1, Table 2, Table 3), one has to take into account: (a) the relatively low number of patients included in several trials, (b) the retrospective nature of (most) studies with often inconsistent histological grading, (c) unknown MGMT methylation status in several studies, and (d) the chance of bias due to the use of chemotherapy or monoclonal antibodies in some regimens. The role of chemotherapy either during the initial treatment course or in the salvage setting becomes increasingly important. The use of chemotherapy or other agents can influence the outcome, and may also play a role in the incidence rate of radiation late effects, like radiation necrosis.

#### 3.4.1. Patients Re-Irradiated with Conventional Radiotherapy

Niyazi *et al*. [15] delivered concurrent chemotherapy at the primary treatment using temozolomide in 53.3% of the patients and applied bevacizumab (a humanized monoclonal antibody against vascular endothelial growth factor) in 66.7% of the patients during re-irradiation. Imaging revealed a maximum of two patients with changes compatible with radiation necrosis, no histological confirmation was established, nor was the use of bevacizumab in these two patients clearly stated. Clinical side-effects of bevacizumab, according to Common Toxicity Criteria, were wound healing complications grade 4 (n = 1), deep vein thrombosis grade 3 (n = 1) and hypertension grade 2 (n = 1). Survival rate was significantly higher in the group with bevacizumab with a mean survival of 187.4 days after re-irradiation alone, compared to 367.6 days after re-irradiation plus bevacizumab. Two studies [10,11] reported on the use of Lomustine in the re-irradiation setting; however, no information on late side-effects (e.g., radiation necrosis) was provided. Concerning survival, no conclusions can be drawn as none of the trials included only one histological grading (e.g., GBM) alone.

#### 3.4.2. Patients Re-Irradiated with Fractionated Stereotactic Radiotherapy

In this group, of the 16 studies three reported on chemotherapy in the primary setting. Patel *et al*. [31] used various agents such as temozolomide, carmustine, oxaliplatin, irinotecan, and the PCV (procarbazine, lomustine, vincristine) regimen, in the primary setting. During the re-irradiation course no chemotherapy was performed; however, all patients were subsequently treated with agents such as irinotecan, carmustine and lomustine, and monoclonal antibodies like erlotinib and bevacizumab. In the cohort of 10 GBM patients, one mixed tumour/necrosis was found. In the study of Fokas *et al*. in the primary setting (41 of 53 patients) and in the re-irradiation setting (25 of 53 patients), various agents such temozolomide, ACNU/VM-26 (nimustine/teniposide) and PCV were used [27]. No side-effects ≥grade 2 were observed. The median survival after re-irradiation was 9 months. No significant difference between patients receiving chemotherapy at time of recurrence was found (11 months *versus* 8 months, *p* = 0.1466); however, patient numbers are relatively small.

The second trial originates from Italy and consists of a very homogenous study cohort [30]. A total of 36 GBM patients, all receiving concomitant temozolomide during initial radiotherapy, as well as adjuvant temozolomide for 6–12 cycles, were treated at the time of recurrence with FSRT plus concomitant daily TMZ at a dose of 75 mg/m^2^, given 7 days/week from the first day of RT. The median survival for the whole study cohort was 9.7 months. However, there was a clear influence of the MGMT methylation status. The median survival was 11.3 months in methylated patients and 7.9 months in unmethylated patients; the MGMT methylation status was the only independent prognostic factor in the multivariate Cox proportional hazards regression model.

Apart from these two studies, another four report on additional chemotherapy at the time of re-irradiation. A combined radiochemotherapy approach with Paclitaxel was chosen by Lederman *et al*. [19]; after an EQD2_cumulative_ of 104.4 Gy, a necrosis rate of 10% was observed. Another study reported the application of various chemotherapeutic agents with temozolomide in about a third of the patients and no reported radiation necrosis after EQD2_cumulative_ of 108.1 Gy [29]. Grosu *et al*. described a 13% necrosis rate after an EQD2_cumulative_ of up to 127.5 Gy using temozolomide alone [21]; a survival benefit from additional temozolomide, despite the limited number of patients, was observed. However, it should be noted that temozolomide might act as radiosensitizer, as found in experimental studies [48,49,50] and, obviously, a EQD2_cumulative_ of 100 Gy should not be not exceeded. Gutin *et al*. applied bevacizumab at time of re-irradiation [28]. Median overall survival was 12.5 months for glioblastoma patients; no brain necrosis was observed. Clinical side-effects of bevacizumab were wound healing problems (n = 1), CNS intratumoural haemorrhage (n = 1) and bowel perforation (n = 1) [28]. Those studies that included only GBM patients showed survival ranging from 7–9 months without chemotherapy at time of re-irradiation, and 7.5–9.7 months by applying chemotherapy at re-irradiation. No definite conclusions can be drawn from these data.

#### 3.4.3. Patients Re-Irradiated with Stereotactic Radiosurgery

In three of seven studies in this group (Table 3), chemotherapy of various regimens was used at the initial treatment course [31,32,35]. No concomitant chemotherapy was reported at time of SRS; however, Patel *et al*. [31] subsequently initiated salvage chemotherapy using agents like irinotecan, carmustine, and lomustine, and monoclonal antibodies like erlotinib and bevacizumab. Survival rates described after SRS in GBM patients were within the range reported in the FSRT group, *i.e*., 5.3–10 months after re-irradiation.

It can be concluded that, in order to draw firm conclusions, it is necessary for future prospective studies to include only one type of histology/grading (*i.e*., GBM patients only), one type of additional agent, and the MGMT promoter methylation status at the primary treatment and re-irradiation stage, as well as the validated neurotoxicity scoring.

## 4. Experimental Section

After a comprehensive search (January 1996 to July 2011) 30 brain re-irradiation studies were identified [51]. The keywords included reirradiation, brain tumours, glioma, GBM, external radiotherapy, fractionated stereotactic radiotherapy (FSRT), stereotactic radiosurgery (SRS) and side-effects. Due to the retrospective character of this analysis in most cases only the ‘median’ physical dose and “median” time interval could be considered. Studies in which re-irradiation was combined with chemotherapy are indicated, as well as one study using hyperbaric hyperoxygenation as radiosensitizer [24]. Brachytherapy studies were not included in this analysis.

To enable calculation of the cumulative Equivalent Total Dose (EQD2) only papers with clearly stated median physical dose of the initial radiation treatment are included (Table 1, Table 2, Table 3). The EQD2 represents the total dose if applied in fractions of 2 Gy [7]. The cumulative EQD2 is defined as the sum of the EQD2 of the initial irradiation course and the EQD2 of the re-irradiation course (EQD2_cumulative_ = EQD2_initial_ + EQD2_re-irradiation_). Furthermore, only patients with reported clinically symptomatic necrosis could be considered. It should be realized that severity of symptoms due to focal necrosis is not only based on the size but also on the location of the injury. If the necrotic volume is small and does not include regions like motor cortex or the brain stem, this damage might be unobserved and remain clinically asymptomatic. On the other hand, necrosis of the same size located in one of those sensitive regions can lead to significant morbidity including seizures, symptoms of increased cranial pressure and neuroanatomic-specific symptoms [52,53]. Also, radionecrosis is often difficult to distinguish from tumour recurrence or progression, even when using functional examinations like MRS, PET or SPECT [54]. For details on histopathological and pathophysiologal characteristics of cerebral necrosis, diagnosis and treatments, we refer to Barani and Sneed [53].

In the present analysis, the tolerance dose was defined as the maximum radiation dose that can be tolerated by the normal brain tissue included in the treatment field, or the biological dose that does not induce any irreversible late radiation toxicity. Clinically or histopathologically confirmed brain necrosis, as well as mixed tumour recurrence/necrosis, were considered as “necrosis” due to irradiation beyond the tolerance of the normal brain tissue. Most studies were not designed to measure late toxicity and patients were not actively assessed for neurotoxicity, which might underestimate the incidence of these effects. In Figure 1, only those studies actually reporting on late radiation-induced necrosis are included.

Radiobiological model calculations were performed using the linear quadratic model assuming complete repair between subsequently applied high-dose rate fractions for the clinical studies using conventional radiotherapy (Table 1), FSRT (Table 2), and LINAC-based SRS (Table 3). Treatment regimens were compared using the EQD2 which is based on the Biologically Effective Dose (BED) concept. The BED was calculated with the linear quadratic model [6,7], according to the following formulae: BED = nd (1 + d/[α/β]) [Gy] with d = fraction dose [Gy], n = number of fractions, nd = D = total physical dose [Gy] and the α/β parameter [Gy]. An α/β ratio of 2 Gy was selected for the late responding normal brain [55]. BED values were converted to an Equivalent total dose delivered in 2 Gy fractions (EQD2) using the formula: EQD2 = BED/(1 + d/α/β), which, at fraction size d of 2 Gy and an α/β ratio of 2 Gy = BED/(1 + 2/2) = BED/2. The linear-quadratic model might be less accurate in the high single-dose range, see Discussion (section 3.1). Graphs were prepared using GraphPad (GraphPad Prism 5.01, Software Inc., San Diego, CA, USA). The correlation analysis (Figure 1) was performed using the nonparametric Pearson correlation test, and two-tailed p-values were calculated.

## 5. Conclusions

Radiation-induced normal brain tissue necrosis was found to occur at EQD2_cumulative_ beyond 100 Gy. The applied re-irradiation dose and EQD2_cumulative_ were found to increase with a change in irradiation technique from conventional to conformal techniques like FSRT, to radiosurgery re-treatment, without increasing the probability of normal brain necrosis. No effect was noticed related to the time interval between the initial and re-irradiation exposure. The mean time interval remarkably decreased with conformality of the re-irradiation therapy. Because of the uniformity of the initial radiation treatment, which is generally a standard regimen of 60 Gy in 2 Gy fractions, our analysis is not conclusive with regard to a likely dependence of tissue recovery on the level of the initial dose.

Finally, in view of the relatively high long-term recovery capacity of the normal human brain, the relatively fast recovery kinetics and tolerance of small treatment volumes to a relatively high total dose, this analysis tends to support further escalation of the target dose in small and well defined recurrences. Because of its beneficial dose distribution, particle irradiation seems to be the superior treatment modality to precisely apply high radiation doses to small target volumes, and should therefore be considered when planning future clinical trials.
